# Evaluation of 6′-Sialyllactose Sodium Salt Supplementation to Formula on Growth and Clinical Parameters in Neonatal Piglets

**DOI:** 10.3390/nu12041030

**Published:** 2020-04-09

**Authors:** Marcia H. Monaco, Dae Hee Kim, Rit B. Gurung, Sharon M. Donovan

**Affiliations:** 1 Department of Food Science and Human Nutrition, University of Illinois, Urbana, IL 61801, USA; monaco@Illinois.edu; 2 GeneChem Inc., Daejeon 34025, Korea; daeheekim@genechem.co.kr (D.H.K.); rgchem@genechem.co.kr (R.B.G.)

**Keywords:** human milk oligosaccharides, 6′-sialyllactose, infant formula, piglet

## Abstract

Oligosaccharides are complex, non-digestible glycans found in large abundance in human milk. The abundance and the profile of bovine milk oligosaccharides and bovine milk based in infant formula differ from those in human milk. Recently, some human milk oligosaccharides (HMOs) have been supplemented to infant formula, however, not all forms have been available in large scale. The objective of the study was to investigate the dose-dependent effects of an enzymatically-synthesized 6′-sialyllactose (6′-SL) sodium salt supplemented to swine milk replacer on growth, hematological parameters, and organ microscopic assessment in our pre-clinical neonatal pig model. Two-day-old male and female pigs (*n* = 47) were provided one of four experimental diets for 21 days. Diets were formulated to contain 0 (CON), 300 (LOW), 600 (MOD), or 1200 (HIGH) mg/L of 6′-SL sodium salt. On days 8 and 22, samples were collected for hematological and histological analyses. Supplemental 6′-SL sodium salt at all doses supported growth and development comparable to those observed in control animals. In addition, serum chemistries, hematology, and organ microscopic structure were unaffected by 6′-SL (*p* > 0.05). Thus, addition of enzymatically-synthesized 6′-SL to a milk replacer formula supported growth and clinical outcomes similar to the control formula in the neonatal piglet.

## 1. Introduction

The milk of all species contains a complex mixture of soluble oligosaccharides that differ in their size, structure, and specific linkages. The backbone is lactose, which can be fucosylated or sialylated, in either alpha or beta configurations. The main milk oligosaccharides are 2′-fucosyllactose (2′-FL), 3-fucosyllactose (3-FL), 3′-sialyllactose (3′-SL), 6′-sialyllactose (6′-SL), and lacto-N-tetraose (LNT) [1]. Human milk is distinct from the milk of other species in its high concentration (5–15 g/L) and structural diversity (>150 forms) of oligosaccharides (HMO), of which 12–14% are sialylated [2,3]. A total of 30 sialylated HMOs have been identified [4], of which 6′- SL and 3′-SL are among the most abundant.

Concentrations of 3′- and 6′-SL in human milk are higher in colostrum than mature milk. Samuel and colleagues measured HMO concentrations at 6 time points over the first 4 months of lactation in a sample of ~300 mothers [5]. At 2-days of age, the 3′-SL concentration was 254 ± 90 mg/L (mean ± SD; range 100–599), then dropped to 149 ± 38 mg/L (range 74–322) by day 13 of lactation, after which the 3′-SL concentration was relatively stable at 129–141 mg/L (range 63–314) between 30 and 120 days of lactation [5]. At 2-days of age, the 6′-SL concentration was 543 ± 166 mg/L (mean ± SD; range 168-1096). The 6′-SL concentration slightly increased to 649 ± 189 mg/L (range 166–1310) by day 13 of lactation, after which the 6′-SL concentrations steadily declined to 465, 231, 151 and 101 mg/L at 30, 60, 90, and 120 days of lactation, respectively [5].

Most infant formulas are produced with bovine milk as the starting material, which contains much lower concentrations of milk oligosaccharides than human milk; 1–2 g/L in bovine colostrum and 100 mg/L in mature bovine milk [6]. A total of 30–50 bovine milk oligosaccharides (BMO) structures have been reported in bovine milk and colostrum [7]. Human and bovine milk share at least 10 common structures, of which 3′-SL and 6′-SL comprise a largest percentage of the BMO [7,8]. Earlier reports quantified the levels of oligosaccharide bound sialic acid in mature bovine milk at 33 mg/L and unsupplemented infant formula at 38 mg/L [9]. Thus, formula-fed infants are receiving less dietary sialylated oligosaccharides than breastfed infants. Sialylated oligosaccharides have numerous biological functions in the newborn, such as anti-infective activity, immune function, gut maturation, and bifidogenic activity [10]. Furthermore, sialic acid is a key component of brain gangliosides and an essential nutrient in the development of the brain, synaptic connection, and memory formation [11]. Thus, there is an interest in supplementing infant formula with 3′-SL and 6′-SL.

Some studies have investigated supplemental sialylated HMO using whey fractions from bovine milk that are enriched with 3′-SL and 6′-SL, but also contain lactose and other whey components [12]. Having pure forms of 3′-SL and 6′-SL would enable precise supplementation of these HMOs in terms of concentrations and ratios. Enzymes and microbes are natural catalysts for oligosaccharide synthesis and have become the most common biological method used for the production of trisaccharides, such as sialyllactose [13]. The 6′-SL sodium salt tested herein was enzymatically synthesized using a strain of beta-D-galactosidase deficient *Escherichia coli* (*E. coli*) BW25113 and a series of enzymes, as described by Gurung and coworkers [14]. The objective of this study was to assess the safety of 6′-SL sodium salt incorporation to a milk replacer at levels that closely correspond to those present in human milk in the preclinical piglet model. The findings of this study showed that incorporation of 6′-SL sodium salt to formula supported normal growth and clinical parameters in young pigs.

## 2. Materials and Methods

### 2.1. Test Ingredient

The test ingredient, 6′-SL sodium salt (>98% purity), was produced by enzymatic synthesis and was provided in powdered form by GeneChem Inc. (Daejeon, South Korea). The 6′-SL sodium salt was synthesized using a set of enzymes derived from an *E. coli* K-12 strain (beta-D-galactosidase deficient *E. coli* BW25113). The 6′-SL sodium salt preparation also contained lactose (0.09%) and N-acetylneuraminic acid (1.13%) [14].

### 2.2. Animal Care and Housing

The study design and animal protocol were identical to that previously described by Monaco et al. [15]. Briefly, two-day old piglets (*n* = 48; an equal proportion of males and females from 12 different litters; 4 piglets per litter) were obtained from Carthage Veterinary Services and were transferred to the AAALAC-approved animal facilities in the Edward R. Madigan Laboratory on the University of Illinois campus. Upon arrival to the animal facility, piglets were randomized to dietary treatments described below, to balance initial body weight and sex. All animal and experimental procedures were in accordance with the National Research Council Guide for the Care and Use of Laboratory Animals [16] and approved by the University of Illinois at Urbana-Champaign Institutional Animal Care and Use Committee (protocol 17076).

### 2.3. Dietary Treatments

Piglets were fed one of four diets with varying amounts of 6′-SL sodium salt. The control diet (CON, *N* = 12) was a commercially available non-medicated sow-milk replacer formula (Advance Liqui-Wean, Milk Specialties Co., Dundee, IL, USA), which was formulated to meet or exceed nutrient requirements for 3 to 5 kg piglets [16]. The remaining dietary treatments used the CON formula supplemented with 6′-SL sodium salt at the following concentrations: 300 mg/L (LOW, *N* = 12), 600 mg/L (MED, *N* = 12), and 1200 mg/L (HIGH, *N*=12). When correcting for the sodium content, these concentrations corresponded to 289.9, 579.8, and 1159.7 mg/L of 6′-SL, respectively. Daily animal care and formula preparation has been described in Monaco et al. [15]. Piglets were housed individually in cages designed to provide piglets easy access to food, roaming room, and additional heat via heating pads. Piglets were weighed each morning prior to feeding to determine milk volume to be dispensed. The formula was reconstituted at a concentration of 183 g/L and piglets were provided 300 and 330 mL/ kg BW on study days 1–5 and 6–21, respectively. Diets were delivered to the piglets 10-times over a period of 24 h via a peristaltic pump. After the 24 h period, any remaining milk was recorded to calculate feed intake. Watery diarrhea over a period of 3 days was observed in one CON piglet, who was removed from the study. Thus, the final number of piglets in the CON group was 11, while all other groups contained 12 animals each.

### 2.4. Sample Collection

On d8 of the study (postnatal day [PND] 10), blood was collected via jugular vein in either plain, K_2_EDTA, or Na Citrate-laced vacuum tubes (BD Biosciences, Franklin Lakes, NJ) to perform chemistry, complete blood count (CBC), and coagulation time analyses, respectively. On d22, animals were sedated with an intramuscular injection of Telazol^®^ (Tiletamine HCl and Zolazepam HCl, 3.5 mg/kg BW each, Pfizer Animal Health, Fort Dodge, IA) and blood was collected via cardiac puncture for chemistries, CBC, and coagulation time analyses 3–6 h after the final feeding. Piglets were then euthanized by an intravenous injection of 86 mg/kg BW sodium pentobarbital (Euthasol; Virbac AH, Inc., Fort Worth, TX). Urine samples for urinalysis, spleen, stomach, kidneys, heart, lungs, intestine, and liver were collected and fixed for analysis as previously described [15].

### 2.5. Microscopic Histological Analysis

A board-certified pathologist with Veterinary Diagnostic Pathology, LLC (Fort Valley, VA), performed microscopic histological analyses on formalin-fixed tissue samples obtained from piglets fed the CON (0 mg 6′-SL/L) and HIGH (1200 mg 6′-SL/L) diets. The slides were examined for tissue-specific lesions, including lymphocyte infiltration, congestion, perfusion, and hemorrhage. Lesions were reported as absent, minimal, mild, moderate, marked, or severe. Microscopic examinations included semiquantitative severity scoring of histopathology lesions and microscopic histologic features. A five-level severity scoring system was employed in which: 0 = absent; 1 = minimal; 2 = mild; 3 = moderate; 4 = marked; and 5 = severe. When uncertainty existed between scoring groups in assigning a value, half values were given.

### 2.6. Hematological Analyses and Urinalysis

Serum chemistries, coagulation time, and CBC analyses and urinalysis followed protocols described in Monaco et al. [15]. Briefly, coagulation time (partial prothrombin time [PT] and activated partial thromboplastin time [aPTT]) were determined using a STA-Compact coagulation analyzer (Diagnostica STAGO, FRANCE). Serum chemistry analyses included concentration of minerals (calcium, phosphorus, magnesium), electrolytes (sodium, potassium, chloride), protein (total protein, albumin, and globulin), metabolites (glucose, total cholesterol, triglycerides, creatinine, urea, total bilirubin, and bicarbonate), the enzymes alkaline phosphatase (ALP), aspartate transaminase (AST), gamma glutamyltransferase (GGT), creatine phosphokinase (CPK), and glutamate dehydrogenase (GLDH) and were performed using an Olympus AU680 chemistry analyzer (Beckman Coulter, Brea, CA, USA). Complete cell blood count (CBC) was performed on CELL-DYN^®^ 3700 (Abbott, Abbott Park, IL, USA) while diagnostic lab trained technicians performed the differential analysis. The variables evaluated in our study were: red blood cells count (RBC), hemoglobin concentration, hematocrit value, mean corpuscular volume (MCV), mean corpuscular hemoglobin (MCH), mean corpuscular hemoglobin concentration (MCHC), and corpuscular hemoglobin concentration mean. Total white blood cell (WBC) count and differential WBC analyses (neutrophils and lymphocytes) were also performed. Platelet indices were analyzed and included platelet count and mean platelet volume (MPV). Urinalysis was performed with the CLINITEK Advantus^®^ Urine Chemistry Analyzer (Siemens Healthcare, Kemnath, Germany), which provided readings of pH, protein, glucose, ketones, bilirubin, and blood. Specific gravity was measured on a refractometer and analyses of urine sediments and cells were done microscopically.

### 2.7. Statistical Analysis

Analysis of variance (ANOVA) was conducted using the MIXED procedure of SAS 9.4 (SAS Inst. Inc., Cary, NC, USA) to differentiate dietary treatment effects on young pigs. Depending on the outcome, one of two statistical models were used: 1) data collected at a single time-point (i.e., organ weights, urine analysis, and intestinal content pH) were analyzed by 1-way ANOVA, and 2) data collected from the same animal on more than one occasion (i.e., daily body weights, formula intake, chemistry, CBC, and coagulation time) were analyzed by ANOVA with repeated-measures in which dietary treatment, time, and treatment by time interaction were added to the statistical model. When dietary treatment or time was significant, with no interaction, data were pooled for statistical representation. When no effect of 6′-SL sodium salt treatment was observed, serum chemistry data were combined and analyzed for differences between d8 and d22. Normality was checked by the Shapiro-Wilk test and outliers were identified by a studentized residual procedure where values of >3 or <−3 were considered as potential outliers. Categorical data (urinalysis, CBC, and microscopic histological analysis) were presented in frequency tables. Data was expressed as mean ± standard error of the means (SEM) and statistical significance was defined at *p* < 0.05.

## 3. Results

### 3.1. Growth and Body Weight Gain

Dietary supplementation of 6′-SL sodium salt at the concentrations of 300 (LOW), 600 (MOD), and 1200 (HIGH) mg/L to a milk replacer formula resulted in no significant differences in growth patterns (Supplementary Figure S1A) or total body weight gain (Supplementary Figure S1B) between the treatment groups. Dietary 6′-SL supplementation had no impact on food consumption (mL/kg BW) (Supplementary Figure S2).

### 3.2. Organ Weights and Intestinal Length, Colonic Content pH

Neither absolute (Supplementary Table S1) nor normalized (per kg BW) (Table 1) intestinal length or organ weights were affected by any dose of 6′-SL sodium salt. Similarly, the pH of colonic contents was similar among the treatment groups (Supplementary Table S2).

### 3.3. Organ Microscopic Histological Analyses

Some minimal or mild, and a few marked microscopic findings were detected in the neonatal piglet tissues (Table 2). The brain, mesenteric lymph nodes, gallbladder, and heart did not present any histological anomalies. Most common findings were lymphocyte infiltration of the stomach, and small and large intestine. Marked hepatic glycogen accumulation and colonic lymphoid nodules were observed, however they were found equally in both CON and HIGH groups.

### 3.4. Hematological Analyses and Urinalysis

**Figure 1:**
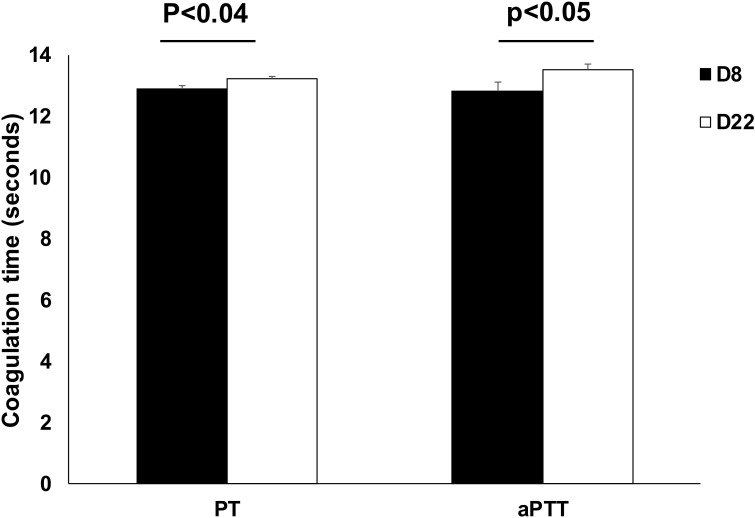
Effect of postnatal age on partial prothrombin time (PT) and activated partial thromboplastin time (aPTT). Values are means ± SEM. These data are also presented in Table 3. Data were combined by time, since treatment effect was not significant.

PT and aPPT were measured on study d8 and d22 to characterize blood coagulation time (Table 3). Administration of 6′-SL at any dose had no effect on PT and aPTT measures, but a time effect was observed as d8 PT and aPTT values were significantly lower than d22 (Figure 1). Blood chemistry values were all within reference ranges at both time points and 6′-SL sodium salt supplementation had no effect on the parameters measured (Supplementary Tables S3–S6, [17,18,19]). Piglet age (d8 vs d22) significantly affected several serum chemistry parameters (Table 4). Serum magnesium, sodium, potassium, chloride, triglycerides, total protein, globulin, ALP, and anion gap were significantly higher on d8 than d22, whereas phosphorus, sodium:potassium ratio, glucose, albumin, albumin:globulin ratio, creatine, and BUN were higher on d22 than d8.

CBC analysis with differential were performed on days 8 and 22 and all values were within expected ranges. There was no statistically significant treatment effect on the parameters RBC, hemoglobin, hematocrit, MCV, MCH, MCHC, platelet, MPV, WBC, neutrophils, and lymphocytes, (Supplementary Tables S7 and S8). Age had an effect on RBC, hemoglobin, hematocrit, MCV, MCH, MPV, and neutrophils (d8 > d22) and monocytes and monocyte counts (d22 > d8) (Table 5). Urine specific activity and pH did not differ among the treatment groups (data not shown). Red blood cells were observed in 54% to 75% of the samples, while white blood cells were found in 66% to 91% of the samples (Supplementary Table S9). Epithelial cells were found in most samples, while bacteria and crystals were found in a small proportion of the samples. These findings were independent on the presence of 6′-SL sodium salt.

## 4. Discussion

Advances in metabolic bioengineering have permitted the scalability of HMOs production for their use as a food ingredient. Large quantities of HMOs are now available, which will facilitate their addition to infant formula. However, the safety and efficacy of these novel components must be determined prior to their use in infant diets. The present study demonstrated that consumption of formula supplemented with 6′-SL sodium salt was well tolerated and supported growth compared to that observed in piglets receiving unsupplemented formula during the first 3 weeks of life. The doses of 6′-SL utilized in the study were comparable to those found in human milk, and reflected concentrations found in the colostrum (range 250–1300 mg/L; MOD and HIGH) and mature milk (range 170–500 mg/L; LOW) [5,9]. In addition, none of the 6′-SL doses tested had produced adverse effects on hematological, chemical, and histological parameters and were comparable to sow-reared and artificially-reared piglets at similar ages [17,21].

Oligosaccharides are increasingly recognized as bioactive components of milk, and are believed to have an impact on the early development of the neonate [22]. Sialic acid-containing oligosaccharides have been implicated in inhibition of pathogen adhesion to host cells, immune system maturation, brain development, and modulation of the gut microbiota [23,24,25]. For example, 6′-SL was shown to reduce rotavirus (RV) infectivity in vitro at concentration levels of 2 to 6 mg/mL [26]. Additionally, orally supplemented 6′-SL caused a significantly enhanced bacterial clearance in a *Pseudomonas aeruginosa* K—induced pneumonia mouse model [27]. In terms of cognitive development, 6′-SL is an integral component of gangliosides and signaling molecules in the brain, and studies have suggested an association between sialic acid-bound gangliosides and glycoproteins with improved learning ability [28,29]. Jacobi et al. [23] reported that formula supplementation with 2 g/L 6′-SL significantly increased the ganglioside-bound SA content in the corpus callosum of pigs compared to piglets receiving non-supplemented formula. Furthermore, Jacobi et al. [23] demonstrated a prebiotic action of 6′-SL to modulate the gut microbiome [23].

The purpose of this study was to examine the safety of enzymatically-synthesized 6′-SL sodium salt supplementation in various clinical and morphological parameters in the neonatal piglet model. Blood count and chemistry, and coagulation time values were all within ranges expected for pigs. Significant differences were observed for both serum chemistry and coagulation time between study days 8 and 22. These differences were not unexpected, as hematological and biochemical parameters are affected by a variety of factors including age, sex, and breed, and reflect developmental stage of the organs and their metabolism [19,30]. Tissue microscopic evaluation was performed on samples collected from animals fed the CON and HIGH diets. The results indicated inflammatory processes in a few organs in both groups, leading to the conclusion that those findings were independent of 6′-SL supplementation. Others have reported that incidental microscopic findings are occasionally seen in piglets of similar age to those used in our study [21,31]. Another minor finding was the presence of blood cells in urine, which was independent on 6-SL supplementation. A small amount of red blood cells in urine is commonly found when the sample is obtained via cystocentesis [32], thus may reflect the sample collection method.

The data herein show that supplementing formula with 6′-SL sodium salt at concentrations up to 1200 mg/L did not affect formula intake and fecal scores, and growth was similar to those reported in previous studies including a supplementation with enzymatically-synthesized 3′-SL [15]. In addition, previous studies have demonstrated the lack of toxicity in rats when fed 5.0 g 6′-SL sodium salt /kg body weight/day [14], which provides additional evidence of the safety and tolerability of enzymatically-synthesized 6′-SL sodium salt. The efficacy of 6′-SL bioactivity in the piglet was not in the purview of our studies, however, more studies should be conducted to determine whether enzymatically-synthesized sialyllactose compounds can replicate the putative biological functions in the brain and microbiota shown in other studies.

## 5. Conclusions

Tremendous progress has been made in recent years in the area of synthetic oligosaccharide production. Chemoenzymatic methods combine the flexibility of chemical synthesis, and the efficiency and selectivity of enzymatic methods to obtain diverse complex carbohydrates, including sialylated compounds. As such, the availability of enzymatically-synthesized forms of SL will facilitate the supplementation of infant formula to bring its oligosaccharide content and composition in line with human milk. This study demonstrated that the 6′-SL sodium salt tested herein supported normal growth and development of piglets at concentration up to 1200 mg/L. Furthermore, all clinical parameters were within reference ranges and no dose-dependent effects were observed.

## Figures and Tables

**Table 1:** Organ weights, small intestinal length and weight, and large intestinal length presented as value normalized by body weight of piglets fed formula containing various concentrations of 6′-SL sodium salt.

	Concentration of 6′-SL Sodium Salt in Formula			
Variable	CON (0 mg/L)	LOW (300 mg/L)	MOD (600 mg/L)	HIGH (1200 mg/L)
Small intestine length (cm/kg)	129 ± 6.9	125 ± 3.0	131 ± 4.9	127 ± 3.5
Small intestine weight (g/kg)	40 ± 1.0	39 ± 1.8	42 ± 2.0	41 ± 2.4
Large intestine length (cm/kg)	24 ± 1.0	24 ± 0.6	24 ± 0.9	24 ± 1.1
Brain (g/kg)	6.1 ± 0.2	5.9 ± 0.2	6.0 ± 0.2	5.9 ± 0.2
Kidneys (g/kg)	6.9 ± 0.2	7.2 ± 0.2	7.2 ± 0.2	7.3 ± 0.2
Spleen (g/kg)	2.2 ± 0.1	2.3 ± 0.1	2.3 ± 0.1	2.3 ± 1.1
Heart (g/kg)	5.7 ± 0.1	5.9 ± 0.1	5.7 ± 0.1	5.7 ± 0.1
Stomach (g/kg)	5.3 ± 0.2	5.2 ± 0.2	5.6 ± 0.2	5.6 ± 0.2
Liver (g/kg)	34 ± 1.2	32 ± 1.3	33 ± 1.0	33 ± 1.4
Lung (g/kg)	12 ± 0.5	12 ± 0.5	12 ± 0.4	13 ± 0.5

Values are means ± SEM. 6′-SL sodium salt supplementation had no effect on organ weights and intestinal length normalized by body weight.

**Table 2:** The incidence of microscopic histological findings in organs of piglets fed formula alone or formula + 1200 mg /L of 6′-SL sodium salt. Data are shown as the number of animals displaying the characteristic. A five-level severity scoring system was employed in which: 0 = absent; 1 = minimal; 2 = mild; 3 = moderate; 4 = marked; and 5 = severe.

Organ	CON (0 mg 6′-SL/L)	HIGH (1200 mg 6′-SL/L)
	N = 11	N = 12
Stomach, pylorus: lymphocyte infiltration		
Minimal	4	2
Mild	7	8
Stomach, glandular: lymphocyte infiltration		
Minimal	3	4
Mild	8	7
Moderate	0	1
Spleen: congestion/perfusion		
Minimal	0	2
Mild	3	4
Moderate	8	5
Marked	0	1
Liver: extramedullary hematopoiesis		
Minimal	6	3
Mild	0	4
Liver: vacuolar change (glycogen accumulation)		1
Mild	2	6
Moderate	5	5
Marked	4	
Kidney: hemorrhage		
Minimal	1	3
Mild	4	6
Moderate	1	0
Kidney: congestion/perfusion		
Minimal	0	2
Mild	10	9
Moderate	1	1
Duodenum: lymphocyte infiltration		
Mild	8	9
Moderate	3	3
Jejunum: lymphocyte infiltration		12
Mild	11	12
Jejunum: congestion/perfusion		
Minimal	4	1
Mild	7	9
Moderate	0	2
Ileum: congestion/perfusion		
Minimal	2	1
Mild	7	11
Moderate	1	0
Cecum: lymphocyte infiltration		
Minimum	0	4
Mild	9	8
Moderate	2	0
Ascending colon: lymphocyte infiltration	11	12
Mild	11	12
Ascending colon: congestion/perfusion		
Minimal	1	4
Mild	10	7
Moderate	0	1
Ascending colon: lymphoid nodule		
Mild	2	0
Moderate	4	5
Marked	4	5
Descending colon: lymphocyte infiltration		
Mild	8	7
Moderate	3	5

**Table 3:** Partial prothrombin time (PT) and activated partial thromboplastin time (aPTT) measured in the serum of piglets fed formula containing various concentrations of 6′-SL sodium salt on study days 8 and 22.

		Concentration of 6′-SL Sodium Salt in Formula			
Variable	Reference Ranges ^2^	CON (0 mg/L)	LOW (300 mg/L)	MOD (600 mg/L)	HIGH (1200 mg/L)
**Day 8**					
PT (sec)	9.3–13.3	12.8 ± 0.23	13.3 ± 0.15	12.7 ± 0.29	12.8 ± 0.20
aPTT (sec)	12.3–17.8	12.8 ± 0.62	13.4 ± 0.33	13.2 ± 0.38	12.0 ± 0.52
**Day 22**					
PT (sec)	9.3–13.3	13.1 ± 0.17	13.1 ± 0.24	13.5 ± 0.20	13.1 ± 0.23
aPTT (sec)	12.3–17.8	13.6 ± 0.36	13.2 ± 0.38	13.8 ± 0.53	13.4 ± 0.57

Values are means ± SEM. 6′-SL sodium salt supplementation had no effect on PT or aPTT activity. ^2^ Range from 20-week-old Yucatan micropigs [20].

**Table 4:** Serum chemistry variables that differed significantly between day 8 and day 22 independent of 6′-SL sodium salt supplementation.

Variable	Units	Day 8	Day 22
Minerals ^2^			
Phosphorus	mg/dL	10.4 ± 0.08	10.8 ± 0.09 *
Magnesium	mg/dL	3.0 ± 0.05 ^†^	2.6 ± 0.04
Electrolytes ^2^			
Sodium	mmol/L	142 ± 0.2 ^†^	141 ± 0.3
Potassium	mmol/L	7.5 ± 0.1 ^†^	5.9 ± 0.1
Sodium:Potassium		19 ± 0.3	24 ± 0.6 *
Chloride	mmol/L	105 ± 0.3 ^†^	104 ± 0.3
Metabolites ^2^			
Glucose	mg/dL	125 ± 2.5	154 ± 1.7 *
Triglycerides	mg/dL	56 ± 4.9 ^†^	34 ± 2.7
Protein ^2^			
Total Protein	g/dL	4.2 ± 0.06 ^†^	3.8 ± 0.06
Albumin	g/dL	1.6 ± 0.03	2.5 ± 0.05 *
Globulin	g/dL	2.6 ± 0.06 ^†^	1.3 ± 0.03
Albumin:Globulin		0.6 ± 0.02	2.0 ± 0.05 *
Enzymes ^3^			
ALP	U/L	1195 ± 51.1 ^†^	480 ± 26.3
Kidney function ^3^			
Creatinine	mg/dL	0.8 ± 0.02	0.9 ± 0.02 *
BUN (Urea)	mg/dL	3.5 ± 0.2	8.1 ± 0.2 *
Acid:Base status ^3^			
Anion Gap		21 ± 0.2 ^†^	17 ± 0.4

Abbreviations: ALP, alkaline phosphatase; BUN, blood urea nitrogen Values are means ± SEM; Data were combined by time point, since treatment was not significant ^2^ This data also appears in Supplementary Tables S3 and S5. ^3^ This data also appears in Supplementary Tables S4 and S6 ^†^ Day 8 > day 22 (*p* < 0.05) * Day 22 > day 8 (*p* < 0.05).

**Table 5:** Cell blood count variables that differed significantly between day 8 and day 22 independent of 6′-SL sodium salt supplementation.

Variable	Units	Day 8	Day 22
RBC	x 10^6^/µL	5.37 ± 0.1 ^†^	5.15 ± 0.1
Hemoglobin	g/dL	10.4 ± 0.1 ^†^	9.37 ± 0.2
Hematocrit	%	33.6 ± 0.4 ^†^	30.3 ± 0.7
MCV	fl	66.0 ± 0.5 ^†^	59.9 ± 0.1
MCH	pg	19.3 ± 0.1 ^†^	18.3 ± 0.1
MPV	fl	9.66 ± 0.2 ^†^	8.82 ± 0.2
Neutrophils	%	43.0 ± 1.4 ^†^	30.7 ± 1.7
Monocytes	%	3.37 ± 0.3	9.77 ± 0.7 *
Neutrophil count	x 10^3^/µL	5.16 ± 0.3 ^†^	3.11 ± 0.2
Monocyte count	x 10^3^/µL	0.41 ± 0.04	1.08 ± 0.1 *

Abbreviations: RBC, red blood cells; MCV, mean corpuscular volume; MCH, mean corpuscular hemoglobin; MPV, mean platelet volume. Values are means ± SEM. ^†^ Day 8 > day 22 (*p* < 0.05) * Day 22 > day 8 (*p* < 0.05).

## Supplementary Materials

The following are available online at https://www.mdpi.com/2072-6643/12/4/1030/s1, Figure S1: Growth curves of piglets fed formula containing various concentrations of 6′sialyllactose, Figure S2: Mean formula consumption containing various concentrations of 6′sialyllactose, Table S1: Body weight and absolute small intestinal weight and length and organ weights of piglets fed formula containing various concentrations of 6′-SL sodium salt., Table S2: pH of colonic and cecal contents of piglets fed formula containing various concentrations of 6′-SL sodium salt., Table S3: Serum minerals, electrolytes, metabolites, and proteins measured in the serum of piglets fed formula containing various concentrations of 6′-SL sodium salt on study day 8., Table S4: Enzymes, indicators of renal and liver function and acid:base balance measured in the serum of piglets fed formula containing various concentrations of 6′-SL sodium salt on study day 8., Table S5: Serum minerals, electrolytes, metabolites, and proteins measured in the serum of piglets fed formula containing various concentrations of 6′-SL sodium salt on study day 22., Table S6: Enzymes, indicators of renal and liver function and acid:base balance measured in the serum of piglets fed formula containing various concentrations of 6′-SL sodium salt on study day 22., Table S7: Complete blood count and differential analysis measured in the serum of piglets fed formula containing various concentrations of 6′-SL sodium salt on study 8., Table S8: Complete blood count and differential analysis measured in the serum of piglets fed formula containing various concentrations of 6′-SL sodium salt on study 22., Table S9: Number of urine samples (% total) containing any glucose, blood, white blood cells, red blood cells and epithelial cells, bacteria and crystals in piglets fed formula containing various concentrations of 6′-SL sodium salt on study 22.
