# Supplementary material for: Evaluation of 6′-Sialyllactose Sodium Salt Supplementation to Formula on Growth and Clinical Parameters in Neonatal Piglets
Source: Nutrients. 2020 Apr 9;12(4):1030. doi: 10.3390/nu12041030 (PMC7230961; doi:10.3390/nu12041030)
Supplement: Supplementary file 1 [file nutrients-12-01030-s001.pdf]

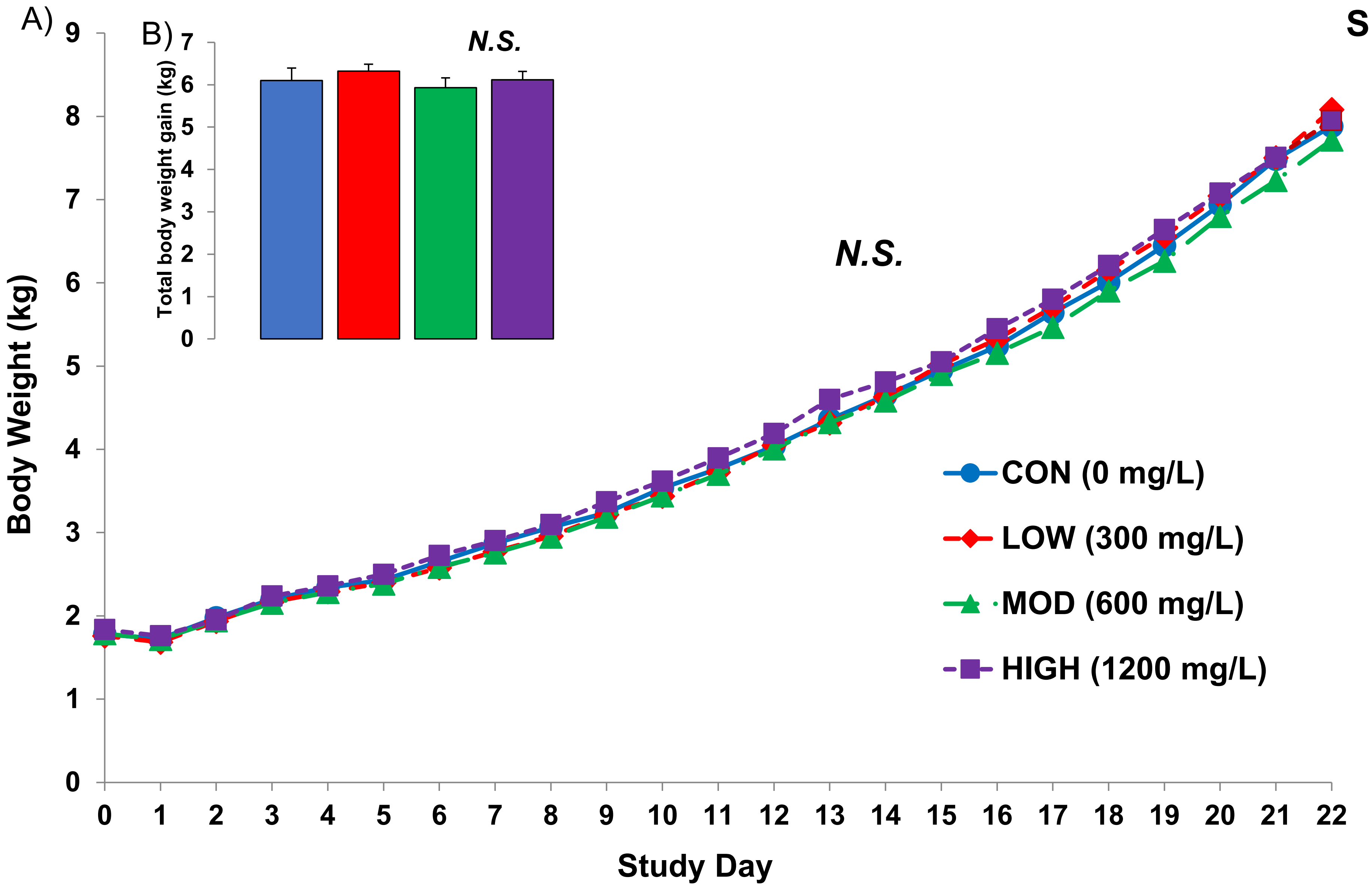

Supplemental Figure 1. Growth curves of piglets fed formula containing various concentrations of 6'sialyllactose from study days 0 to 22 (postnatal days 2-24). B) Total weight gain (kg) calculated as the difference in weights on study days 0 and 22. Values are means (A) or means ± SEM (B). N.S – not significant, p>0.05.

Supplemental Figure 2

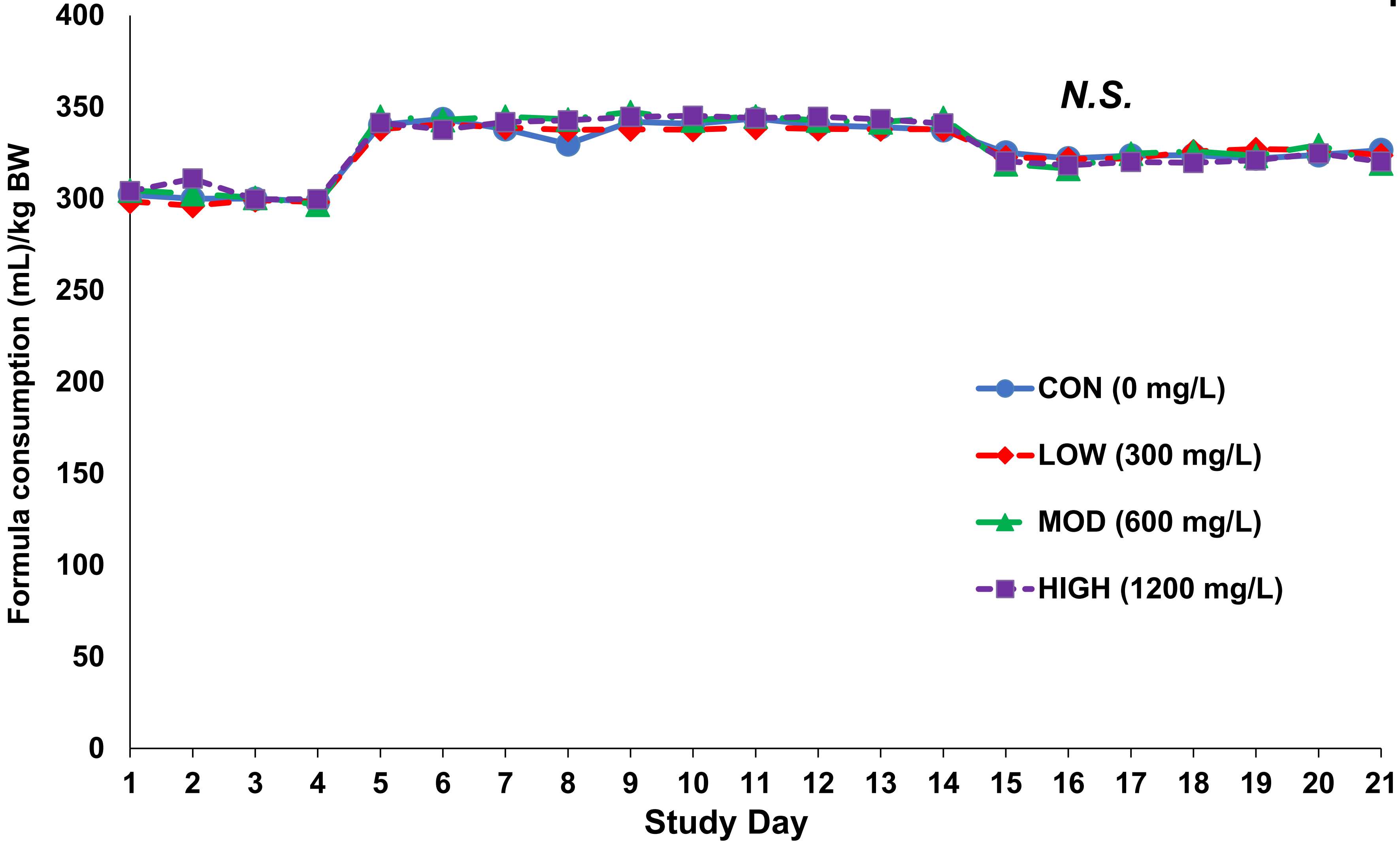

Supplemental Figure 2. Mean formula consumption containing various concentrations of 6'sialyllactose from study days 0 to 22 (postnatal days 2-24). Formula volume was normalized by body weight (kg). N.S – not significant, p>0.05.

Supplemental Table 1. Body weight and absolute small intestinal weight and length and organ weights of piglets fed formula containing various concentrations of 6'-SL sodium salt[1]

Variable	Concentration of 6'-SL sodium salt in Formula			
	CON (0 mg/L)	LOW (300 mg/L)	MOD (600 mg/L)	HIGH (1,200 mg/L)
Small Intestine length (cm)	1033 ± 38	1023 ± 181	1039 ± 26	1038 ± 24
Small Intestine weight (g)	323 ± 15	320 ± 11	328 ± 9.3	330 ± 16
Large intestine length (cm)	190 ± 5.1	197 ± 6.1	193 ± 5.7	196 ± 9.7
Brain (g)	48.9 ± 0.8	48.4 ± 0.7	47.6 ± 1.1	48.0 ± 0.7
Kidneys (g)	56.2 ± 2.9	59.5 ± 2.2	57.8 ± 2.8	59.2 ± 1.5
Spleen (g)	18.1 ± 1.5	18.7 ± 1.1	18.7 ± 1.3	18.8 ± 1.0
Heart (g)	46.1 ± 2.1	45.8 ± 2.0	45.6 ± 1.6	46.6 ± 1.1
Stomach (g)	43.9 ± 2.1	42.9 ± 1.1	44.3 ± 1.1	45.5 ± 1.2
Liver (g)	275 ± 14	259 ± 9.1	267 ± 10	272 ± 11
Lung (g)	100 ± 3.1	102 ± 3.4	99.8 ± 3.9	108 ± 3.4

[1]Values are as means ±SEM.

Supplemental Table 2. pH of colonic and cecal contents of piglets fed formula containing various concentrations of 6'-SL sodium salt[1]

**Diets**	**Ascending Colon**	**Descending Colon**	**Cecum**
**CON (0 mg/L)**	6.29 ± 0.09	6.93 ± 0.08	6.33 ± 0.10
**LOW (300 mg/L)**	6.37 ± 0.07	7.00 ± 0.09	6.49 ± 0.08
**MOD (600 mg/L)**	6.39 ± 0.08	6.99 ± 0.10	6.39 ± 0.11
**HIGH (1,200 mg/L)**	6.44 ± 0.09	6.96 ± 0.08	6.32 ± 0.06

[1]Values are means ± SEMs

Supplemental Table 3. Serum minerals, electrolytes, metabolites and proteins measured in the serum of piglets fed formula containing various concentrations of 6'-SL sodium salt on study day 8[1]

Variable	Units	Reference ranges[2]	Concentration of 6´SL sodium salt in Formula			
			CON (0 mg/L)	Low (300 mg/L)	MOD (600 mg/L)	HIGH (1200 mg/L)
Minerals						
Calcium	mg/dL	7.1-11.6	10.8 ± 0.2	10.5 ± 0.2	10.5 ± 0.1	10.7 ± 0.1
Phosphorous	mg/dL	5.3-9.6	10.6 ± 0.2	10.2 ± 0.1	10.6 ± 0.2	10.4 ± 0.2
Magnesium	mg/dL	2.7-3.7	3.0 ± 0.1	3.1 ± 0.1	3.0 ± 0.1	3.0 ± 0.1
Electrolytes						
Sodium	mmol/L	135-150	142 ± 0.4	142 ± 0.5	141 ± 0.5	142 ± 0.6
Potassium	mmol/L	4.4-6.7	7.6 ± 0.2	7.4 ± 0.3	7.3 ± 0.3	7.5 ± 0.1
Sodium:Potassium			19 ± 0.6	20 ± 0.7	20 ± 0.8	19 ± 0.4
Chloride	mmol/L	94-106	104 ± 0.5	105 ± 0.7	105 ± 0.7	105 ± 0.5
Metabolites						
Glucose	mg/dL	85-150	127 ± 4.6	126 ± 4.8	125 ± 4.4	125 ± 6.5
Cholesterol total	mg/dL	78-116	77 ± 5.1	68 ± 2.6	70 ± 4.8	77 ± 4.2
Triglycerides	mg/dL	29-80	66 ± 12	52 ± 7.8	46 ± 9.2	59 ± 10
Protein						
Total Protein	g/dL	3.7-4.8	4.2 ± 0.2	4.0 ± 0.1	4.2 ± 0.2	4.2 ± 0.1
Albumin	g/dL	1.9-3.9	1.6 ± 0.04	1.5 ± 0.1	1.7 ± 0.1	1.6 ± 0.1
Globulin	g/dL	1.9-3.9	2.6 ± 0.2	2.5 ± 0.1	2.6 ± 0.2	2.6 ± 0.1
Albumin:Globulin			0.7 ± 0.1	0.6 ± 0.04	0.7 ± 0.1	0.6 ± 0.03

[1]Values are means ± SEM. [2] Ranges from 30-day-old pigs (Ventrella et al., 2017)

Supplemental Table 4. Enzymes, indicators of renal and liver function and acid:base balance measured in the serum of piglets fed formula containing various concentrations of 6´-SL sodium salt on study day 8[1]

Variable	Units	Ref ranges[2]	Concentration of 6´-SL sodium salt in Formula			
			CON (0 mg/L)	Low (300 mg/L)	MOD (600 mg/L)	HIGH (1200 mg/L)
Enzymes						
ALP	U/L	110-1292	1185 ± 81	1251 ± 125	1188 ± 102	1148 ± 97
AST	U/L	13-65	32 ± 2.9	33 ± 3.8	42 ± 6.3	42 ± 3.9
GGT	U/L	10-60[3]	47 ± 3.6	51 ± 4.9	48 ± 2.8	52 ± 5.9
CPK	U/L	153-5427[4]	220 ± 29	340 ± 104	608 ± 203	429 ± 98
GLDH	U/L		1.3 ± 0.2	1.5 ± 0.4	2.0 ± 0.3	2.3 ± 0.3
Kidney function						
Creatinine	mg/dL	0.51-1.39	0.82 ± 0.05	0.85 ± 0.04	0.79 ± 0.03	0.80 ± 0.06
BUN (Urea)	mg/dL	4-39	3.7 ± 0.4	3.0 ± 0.3	3.6 ± 0.4	3.8 ± 0.3
Liver function						
Total Bilirubin	mg/dL	0-10[3]	0.17 ± 0.02	0.13 ± 0.01	0.16 ± 0.02	0.18 ± 0.01
Acid:Base status						
Bicarbonate	mmol/L		24 ± 1.6	24 ± 1.6	23 ± 2.2	21 ± 1.9
Anion Gap			21 ± 1.6	20 ± 1.5	21 ± 1.6	23 ± 1.8

Abbreviations: ALP, alkaline phosphatase; AST, aspartate transaminase; CPK, creatine phospholinase; GLDH, glutamate dehydrogenase; GGT, gamma glutamyltransferase; BUN, blood urea nitrogen.

[1]Values are means ± SEM.

[2] Ranges from 30-day-old pigs (Ventrella et al., 2017) unless otherwise stated

[3]Merck Veterinary Manual, Reference Guides (animal age unknown)

[4] Range from 6-week-old cross-bred pigs (Cooper et al., 2014).

Supplemental Table 5. Serum minerals, electrolytes, metabolites and proteins measured in the serum of piglets fed formula containing various concentrations of 6´-SL sodium salt on study day 22 [1]

Variable	Units	Ref ranges[2]	Concentration of 6´-SL sodium salt in Formula			
			CON (0 mg/L)	Low (300 mg/L)	MOD (600 mg/L)	HIGH (1200 mg/L)
Minerals						
Calcium	mg/dL	7.1-11.6	10.7 ± 0.2	10.7 ± 0.1	10.6 ± 0.1	10.9 ± 0.2
Phosphorous	mg/dL	5.3-9.6	10.9 ± 0.2	10.6 ± 0.1	10.6 ± 0.2	11.0 ± 0.2
Magnesium	mg/dL	2.7-3.7	2.5 ± 0.1	2.7 ± 0.05	2.6 ± 0.1	2.6 ± 0.08
Electrolytes						
Sodium	mmol/L	135-150	141 ± 0.6	141 ± 0.6	140 ± 0.5	140 ± 0.6
Potassium	mmol/L	4.4-6.7	6.2 ± 0.2	5.9 ± 0.3	5.7 ± 0.2	6.1 ± 0.3
Sodium:Potassium			23 ± 0.8	25 ± 1.2	25 ± 1.1	24 ± 1.5
Chloride	mmol/L	94-106	105 ± 0.6	105 ± 0.7	105 ± 0.3	104 ± 0.4
Metabolites						
Glucose	mg/dL	85-150	157 ± 5.2	154 ± 2.1	153 ± 2.3	152 ± 3.5
Cholesterol total	mg/dL	78-116	62 ± 5.9	69 ± 2.3	70 ± 3.3	77 ± 5.0
Triglycerides	mg/dL	29-80	40 ± 6.0	28 ± 3.6	33 ± 5.7	37 ± 6.4
Protein						
Total Protein	g/dL	3.7-4.8	3.6 ± 0.2	3.8 ± 0.1	3.8 ± 0.1	3.9 ± 0.1
Albumin	g/dL	1.9-3.9	2.4 ± 0.2	2.6 ± 0.1	2.5 ± 0.1	2.5 ± 0.1
Globulin	g/dL	1.9-3.9	1.3 ± 0.1	1.3 ± 0.1	1.3 ± 0.1	1.4 ± 0.05
Albumin:Globulin			2.2 ± 0.1	2.1 ± 0.1	2.0 ± 0.1	1.9 ± 0.1

[1]Values are means ± SEM. [2] Ranges from 30-day-old pigs (Ventrella et al., 2017).

Supplemental Table 6. Enzymes, indicators of renal and liver function and acid:base balance measured in the serum of piglets fed formula containing various concentrations of 6´-SL sodium salt on study day 22 [1]

Variable	Units	Ref ranges[2]	Concentration of 6´SL sodium salt in Formula			
			**CON (0 mg/L)**	**Low (300 mg/L)**	**MOD (600 mg/L)**	**HIGH (1200 mg/L)**
Enzymes						
ALP	U/L	110-1292	437 ± 71.7	499 ± 47.3	502 ± 54.7	480 ± 41.4
AST*	U/L	13-65	46 ± 8.7	58 ± 17.8	42 ± 6.9	47 ± 10.9
GGT	U/L	10-60[3]	46 ± 5.5	39 ± 3.7	46 ± 7.1	54 ± 8.0
CPK	U/L	153-5427[4]	749 ± 124	913 ± 293	769 ± 129	710 ± 109
GLDH	U/L		1.3 ± 0.2	1.1 ± 0.1	1.1 ± 0.2	1.4 ± 0.2
Kidney function						
Creatinine	mg/dL	0.51-1.39	0.93 ± 0.1	0.90 ± 0.03	0.85 ± 0.05	0.88 ± 0.04
BUN (Urea)	mg/dL	4-39	7.1 ± 0.3	8.2 ± 0.5	8.6 ± 0.3	7.8 ± 0.41
Liver function						
Total Bilirubin*	mg/dL	0-10[3]	0.15 ± 0.02	0.15 ± 0.01	0.13 ± 0.01	0.13 ± 0.01
Acid:Base status						
Bicarbonate*	mmol/L		26 ± 0.6	25 ± 0.6	25 ± 0.8	24 ± 0.6
Anion Gap			16 ± 1.1	17 ± 0.7	17 ± 1.0	18 ± 0.8

Abbreviations: ALP, alkaline phosphatase; AST, aspartate transaminase; CPK, creatine phospholinase; GLDH, glutamate dehydrogenase; GGT, gamma glutamyltransferase; BUN, blood urea nitrogen.

[1]Values are means ± SEM.

[2] Ranges from 30-day-old pigs (Ventrella et al., 2017) unless otherwise stated

[3]Merck Veterinary Manual, Reference Guides (animal age unknown)

[4] Range from 6-week-old cross-bred pigs (Cooper et al., 2014).

6'SL administration had no significant impact on enzymes, indicators of renal and liver function and acid:base balance on d22.

Supplemental Table 7. Complete blood count and differential analysis measured in the serum of piglets fed formula containing various concentrations of 6´-SL sodium salt on study 8[1]

Cell Type	Unit	Reference Ranges[2]	Concentration of 6´SL sodium salt in Formula			
			**CON (0 mg/L)**	**LOW (300 mg/L)**	**MOD (600 mg/L)**	**HIGH (1200 mg/L)**
RBC	x 10^6/µL	4.1-8.2	5.4 ± 0.1	5.3 ± 0.1	5.4 ± 0.1	5.4 ± 0.2
Hemoglobin	g/dL	4.3-13.3	11 ± 0.2	10 ± 0.2	10 ± 0.2	11 ± 0.3
Hematocrit	%	16-41	34 ± 0.9	32 ± 0.7	33 ± 0.8	35 ± 1.1
MCV	fl	34.2-61.3	66 ± 0.6	64 ± 0.9	66 ± 1.3	68 ± 0.9
MCH	pg	9.4-19.8	19 ± 0.1	19 ± 0.2	19 ± 0.2	19 ± 0.3
MCHC	g/dL	26.5-33.6	31 ± 0.4	31 ± 0.5	31 ± 0.4	31 ± 0.3
NRBC	/100WBC		1.7 ± 0.6	2.6 ± 0.6	2.1 ± 0.7	1.5 ± 0.6
Platelet	x 10^3/µL	192-832	561 ± 81	693 ± 75	642 ± 47	678 ± 99
MPV[§]	fl	6.5-12.7	9.7 ± 0.3	9.7 ± 0.6	9.7 ± 0.5	9.6 ± 0.6
WBC	x 10^3/µL	5.6-18.5	11 ± 0.8	11 ± 0.6	13 ± 1.7	12 ± 0.9
Neutrophils	%	10.8-70.6	39 ± 2.4	44 ± 2.7	45 ± 2.4	44 ± 4.1
Lymphocytes	%	26.2-82.9	56 ± 2.4	52 ± 2.8	53 ± 1.2	56 ± 2.6
Monocytes	%	1.4-8.3	3.6 ± 0.7	3.2 ± 0.6	3.9 ± 0.7	2.7 ± 0.6
Eosinophils	%	0-1.9	1.0 ± 0.3	0.6 ± 0.2	0.3 ± 0.2	0.6 ± 0.3
Basophils	%	0-0.9	0.1 ± 0.1	0.5 ± 0.2	0.3 ± 0.1	0.2 ± 0.1
Neutrophil count	x 10^3/µL	0.8-9.7	4.2 ± 0.5	5.0 ± 0.4	6.0 ± 0.5	5.2 ± 0.6
Lymphocyte count	x 10^3/µL	2.7-12.8	5.8 ± 0.4	5.2 ± 0.5	6.7 ± 0.4	6.3 ± 0.6
Monocyte count	x 10^3/µL	0.1-1.1	0.4 ± 0.09	0.4 ± 0.1	0. 5 ± 0.1	0.3 ± 0.08
Eosinophil count	x 10^3/µL	0-0.2	0.1 ± 0.02	0.06 ± 0.02	0.04 ± 0.3	0.08 ± 0.04
Basophil count	x 10^3/µL	0-0.13	0. 01 ± 0.01	0.05 ± 0.02	0.04 ± 0.02	0.02 ± 0.02

Abbreviations: RBC, red blood cells; NRBC, nucleated red blood cell; MCV, mean corpuscular volume; MCHC, mean corpuscular hemoglobin concentration; MPV, mean platelet volume; WBC, white blood cells.

[1]Values are means ± SEMs. [2] From Ventrella et al., 2017

[§]MPV: CON, N=8; LOW, N=8; MOD, N=8; HIGH, N=5.

6'SL administration had no significant impact on CBC variables on d8.

Supplemental Table 8. Complete blood count and differential analysis measured in the serum of piglets fed formula containing various concentrations of 6´-SL sodium salt on study 22[1]

Cell Type	Unit	Reference Ranges[2]	Concentration of 6´-SL sodium salt in Formula			
			CON (0 mg/L)	LOW (300 mg/L)	MOD (600 mg/L)	HIGH (1200 mg/L)
RBC	x $10^6/\mu L$	4.1-8.2	5.4 ± 0.2	5.1 ± 0.2	5.1 ± 0.3	5.1 ± 0.2
Hemoglobin	g/dL	4.3-13.3	9.9 ± 0.3	9.2 ± 0.3	9.1 ± 0.4	9.3 ± 0.3
Hematocrit	%	16-41	33 ± 1.0	30 ± 1.0	30 ± 1.4	29 ± 1.6
MCV	fl	34.2-61.3	60 ± 0.8	60 ± 0.9	59 ± 0.7	60 ± 0.8
MCH	pg	9.4-19.8	18 ± 0.2	18 ± 0.3	18 ± 0.3	18 ± 0.2
MCHC	g/dL	26.5-33.6	31 ± 0.2	31 ± 0.3	31 ± 0.2	31 ± 0.1
NRBC	/100WBC		2.1 ± 0.7	2.0 ± 0.5	1.2 ± 0.5	1.5 ± 0.4
Platelet	x $10^3/\mu L$	192-832	644 ± 83	779 ± 68	730 ± 40	745 ± 42
MPV[§]	fl	6.5-12.7	9.6 ± 0.4	8.1 ± 0.3	8.4 ± 0.3	8.7 ± 0.3
WBC	x $10^3/\mu L$	5.6-18.5	11 ± 0.8	11.± 1.2	10 ± 1.0	11 ± 0.9
Neutrophils	%	10.8-70.6	27 ± 0.1	32 ± 3.4	29 ± 3.8	34 ± 3.4
Lymphocytes	%	26.2-82.9	63 ± 3.7	57 ± 4.9	53 ± 5.4	56 ± 2.7
Monocytes	%	1.4-8.3	11 ± 1.4	9 ± 1.5	8.6 ± 1.5	11 ± 1.0
Eosinophils	%	0-1.9	0.7 ± 0.3	1.3 ± 0.3	0.6 ± 0.3	1.8 ± 0.5
Basophils	%	0-0.9	0.3 ± 0.1	0.1 ± 0.1	0.4 ± 0.2	0.1 ± 0.0.8
Neutrophil count	x $10^3/\mu L$	0.8-9.7	3.0 ± 0.4	2.8 ± 0.4	2.7 ± 0.4	3.8 ± 0.5
Lymphocyte count	x $10^3/\mu L$	2.7-12.8	6.8 ± 0.4	5.7 ± 0.7	5.0 ± 0.7	5.8 ± 0.5
Monocyte count	x $10^3/\mu L$	0.1-1.1	1.2 ± 0.2	1.0 ± 0.2	0. 83 ± 0.1	1.2 ± 0.1
Eosinophil count	x $10^3/\mu L$	0-0.2	0.1 ± 0.05	0.15 ± 0.04	0.06 ± 0.03	0.18 ± 0.05
Basophil count	x $10^3/\mu L$	0-0.13	0. 03 ± 0.02	0.01 ± 0.01	0.02 ± 0.02	0.01 ± 0.01

Abbreviations: RBC, red blood cells; NRBC, nucleated red blood cell; MCV, mean corpuscular volume; MCHC, mean corpuscular hemoglobin concentration; MPV, mean platelet volume; WBC, white blood cells.

[1]Values are means ± SEMs. [2] From Ventrella et al., 2017

[§]MPV: CON, N=4; LOW, N=5; MOD, N=8; HIGH, N=5.

Supplemental Table 9. Number of urine samples (% total) containing any glucose, blood, white blood cells, red blood cells and epithelial cells, bacteria and crystals in piglets fed formula containing various concentrations of
6´-SL sodium salt on study 22[1,2]

Cell Type	Diets (6´-SL Concentration)			
	CON (0 mg/L)	LOW (140 mg/L)	MOD (200 mg/L)	HIGH (500 mg/L)
	N=11	N=12	N=12	N=12
Protein	3 (27.3%)	2 (16.7%)	1 (8.0%)	1 (8.0%)
Glucose	1 (9.1%)	0	0	0
Blood	6 (54.5%)	9 (75.0%)	7 (58.3%)	8 (66.7%)
White blood cells	10 (90.9%)	11 (91.7%)	8 (66.7%)	10 (83.3%)
Red blood cells	11 (100%)	12 (100%)	12 (100%)	11 (91.7%)
Epithelial cells	10 (90.9%)	12 (100%)	12 (100%)	12 (100%)
Bacteria	4 (36.4%)	3 (25.0%)	3 (25.0%)	1 (8.0%)
Crystals	2 (18.2%)	1 (8.3%)	0	0

[1] Categorical characterization of presence of metabolites, cells or crystals consisted of negative (none), rare, trace, few, moderate and many.
[2] Number represented in table reflects number of samples with any level (rare, trace, few, moderate and many).

References

[1] Ventrella, D., Dondi, F., Barone, F., Serafini, F., Elmi, A., Giunti, M., Romagnoli, N., Forni, M., Bacci, M.L. The biomedical piglet: establishing reference intervals for haematology and clinical chemistry parameters of two age groups with and without iron supplementation. BMC Vet. Res. 2017, 13, 23-30. doi: 10.1186/s12917-017-0946-2.

[2] Merck and the Merck Veterinary Manual. Serum biochemical references ranges. http://www.merckvetmanual.com/appendixes/reference-guides/serum-biochemical-reference-ranges.

[3] Cooper, C.A., Moraes, L.E., Murray, J.D., Owens, S.D. Hematologic and biochemical reference intervals for specific pathogen free 6-week-old Hampshire-Yorkshire crossbred pigs. J. Anim. Sc. Biotechnol. 2014, 5, 5. doi: 10.1186/2049-1891-5-5.
